# Nonstructural Proteins Are Preferential Positive Selection Targets in Zika Virus and Related Flaviviruses

**DOI:** 10.1371/journal.pntd.0004978

**Published:** 2016-09-02

**Authors:** Manuela Sironi, Diego Forni, Mario Clerici, Rachele Cagliani

**Affiliations:** 1 Scientific Institute IRCCS E. MEDEA, Bioinformatics, Bosisio Parini, Italy; 2 Department of Physiopathology and Transplantation, University of Milan, Milan, Italy; 3 Don C. Gnocchi Foundation ONLUS, IRCCS, Milan, Italy; Centers for Disease Control and Prevention, UNITED STATES

## Abstract

The *Flavivirus* genus comprises several human pathogens such as dengue virus (DENV), Japanese encephalitis virus (JEV), and Zika virus (ZIKV). Although ZIKV usually causes mild symptoms, growing evidence is linking it to congenital birth defects and to increased risk of Guillain-Barré syndrome. ZIKV encodes a polyprotein that is processed to produce three structural and seven nonstructural (NS) proteins. We investigated the evolution of the viral polyprotein in ZIKV and in related flaviviruses (DENV, Spondweni virus, and Kedougou virus). After accounting for saturation issues, alignment uncertainties, and recombination, we found evidence of episodic positive selection on the branch that separates DENV from the other flaviviruses. NS1 emerged as the major selection target, and selected sites were located in immune epitopes or in functionally important protein regions. Three of these sites are located in an NS1 region that interacts with structural proteins and is essential for virion biogenesis. Analysis of the more recent evolutionary history of ZIKV lineages indicated that positive selection acted on NS5 and NS4B, this latter representing the preferential target. All selected sites were located in the N-terminal portion of NS4B, which inhibits interferon response. One of the positively selected sites (26M/I/T/V) in ZIKV also represents a selection target in sylvatic DENV2 isolates, and a nearby residue evolves adaptively in JEV. Two additional positively selected sites are within a protein region that interacts with host (e.g. STING) and viral (i.e. NS1, NS4A) proteins. Notably, mutations in the NS4B region of other flaviviruses modulate neurovirulence and/or neuroinvasiveness. These results suggest that the positively selected sites we identified modulate viral replication and contribute to immune evasion. These sites should be prioritized in future experimental studies. However, analyses herein detected no selective events associated to the spread of the Asian/American ZIKV lineage.

## Author Summary

Zika virus is mainly transmitted by mosquitoes and is phylogenetically related to other human pathogens (e.g. dengue virus). After the outbreak in South America, the WHO declared that the spread of ZIKV should be regarded as a public health emergency. In fact, growing evidence suggests that ZIKV infection during pregnancy increases the risk of congenital birth defects. Moreover, ZIKV can trigger Guillain- Barré syndrome, a severe neurological disorder characterized by progressive muscle weakness. Evolutionary studies can help identify sites that allow viral adaptation—i.e. that increase viral fitness at least in some conditions. We analyzed the evolution of the polyproteins encoded by ZIKV and by related viruses and identified several sites in nonstructural proteins that were subject to natural selection. Most of these are located in protein regions that mediate interaction with the host immune system or that may regulate viral RNA synthesis. In ZIKV isolates, the NS4B protein was the preferential selection target with three selected residues. Changes at these sites are expected to modulate some aspect of viral fitness, either in mosquitoes or vertebrate hosts. Future studies to clarify the mechanisms of ZIKV pathogenicity should address the role of these sites in the modulation of viral phenotypes.

## Introduction

The *Flavivirus* genus (family *Flaviviridae*) comprises a large number of viral species, many of which are important human pathogens; these include dengue virus (DENV), yellow fever virus (YFV), Japanese encephalitis virus (JEV), West Nile virus (WNV), and the latest emerged pathogen, Zika virus (ZIKV).

ZIKV was first discovered in 1947 in Uganda, in a sentinel rhesus monkey, and subsequently in mosquitoes of the *Aedes* genus. Between 1947 and 2006, only sporadic human cases were reported in Africa and in Southeast Asia, until multiple outbreaks in the Pacific islands occurred. The first sizable outbreak was reported in the Federated States of Micronesia (Yap Island) in 2007, followed by an outbreak in French Polynesia in 2013. In 2014, the epidemic spread to Cook Islands, New Caledonia and Easter Island, and reached South America in late 2014 –early 2015 [1–3]. As of May 18, 2016, sixty countries/territories have reported ZIKV cases (http://www.who.int/emergencies/zika-virus/situation-report/en/).

Although ZIKV infection is often asymptomatic or causes only mild symptoms, the WHO declared that the spread of ZIKV should be regarded as a public health emergency of international concern. In fact, growing evidence suggests that ZIKV infection during pregnancy increases the risk of microcephaly, brain damage, and congenital abnormalities [4–6]. Also, retrospective studies indicated that ZIKV can trigger Guillain-Barré syndrome (GBS), a severe neurological disorder characterized by progressive muscle weakness [7]. Moreover, even if *Aedes* mosquitoes species such as *Aedes aegypti* and *Aedes albopticus* represent the primary vectors for natural transmission, perinatal and congenital infections, as well as sexual transmission and infection through blood transfusion have been recently documented [1].

ZIKV is a member of the Spondweni (SPOV) serocomplex and, like other members of the *Flavivirus* genus, it is a single-stranded positive-sense RNA virus. Its genome consists of about 11,000 nucleotides with two flanking non-coding regions and a single long open reading frame. The encoded polyprotein is co- and post-translationally processed by viral and host proteases to produce three structural (capsid, C; pre-membrane, prM; envelope, E) and seven nonstructural (NS) proteins (NS1, NS2A, NS2B, NS3, NS4A, NS4B, NS5) [8].

Genetic and phylogenetic studies indicated that ZIKV has evolved into 2 major lineages: African and Asian/American, this latter responsible of the recent outbreaks and associated with reports of GBS and fetal malformations [9, 10].

Analysis of ZIKV genomes from microcephaly cases revealed no shared amino acid changes, suggesting that viral genetic features alone are not responsible for fetal abnormalities [2]. Likewise, inspection of amino acid differences between the Asian and African lineages provided no clear indication of viral genetic features that may result in altered virulence or increased pathogenicity, although no functional study of these variants has been performed to date [2]. It was thus proposed that, if the link with GBS and fetal abnormalities is confirmed, factors other than viral genetics, including infection with other viruses or the host genetic background [2], are responsible for these adverse effects. An alternative possibility is that all ZIKV lineages increase the risk of microcephaly and/or GBS, but the association has been previously missed due to the limited size of African outbreaks and to the lack of surveillance programs. Whereas addressing these questions will require extensive epidemiological and clinical surveys, analysis of all ZIKV strains and of their evolution within the wider perspective of closely related flaviviruses can identify positively selected amino acid changes. These latter are expected to entail a functional effect and can therefore be prioritized in further studies of viral pathogenesis. Indeed, evolutionary analyses in WNV have detected positively selected changes that modulate viral phenotypes such as virulence [11] and superinfection exclusion [12].

Herein we investigated the evolution of the viral polyprotein in ZIKV and in related flaviviruses. Results indicate that NS1 was a major selection target during flavivirus speciation and revealed ongoing selection in ZIKV strains in NS4B and NS5.

## Methods

### Sequences and alignments

Coding sequences were retrieved from the NCBI database (http://www.ncbi.nlm.nih.gov/), all flaviviruses analyzed were selected to have full coding sequence information. A list of accession number is reported in S1 Table.

Alignment errors are common when divergent sequences are analyzed and can affect evolutionary inference. Thus, we used PRANK [13] to generate multiple sequence alignments and GUIDANCE2 [14] for filtering unreliably aligned codons (we masked codons with a score <0.90), as suggested [15].

### Substitution saturation and recombination

Substitution saturation was checked using the Xia's index implemented in DAMBE [16, 17]; this test compares an entropy-based index of saturation (I_ss_) with a critical value (I_ss.c_). If I_ss_ is significantly lower than I_ss.c_, sequences have not experienced substitution saturation.

The presence of recombination was assessed using two methods, GARD [18] and Recco [19]. Whereas GARD uses phylogenetic incongruence among segments in the alignment to detect the best-fit number and location of recombination breakpoints, Recco is based on cost minimization and dynamic programming. In GARD, the statistical significance of putative breakpoints is evaluated through Kishino-Hasegawa (HK) tests; breakpoints were considered significant if their *p* value was < 0.05.

The Recco's output includes a *p* value for the whole dataset that, controlling for false positives, provides an indication as to whether a significant amount of recombination is detectable in the whole alignment. We concluded that no substantial recombination was present when the dataset *p* value was >0.05. For alignments showing evidence of recombination in Recco (dataset *p* value <0.05), we considered sequences as recombinants if the number of savings was >20, and the sequence *p* value was <0.001, as suggested [20]. Recombination breakpoints were defined accordingly.

### Evolutionary analyses

Phylogenetic trees were reconstructed using the program phyML with a maximum-likelihood approach, a General Time Reversible (GTR) model plus gamma-distributed rates and 4 substitution rate categories. Branch support was evaluated using a non parametric bootstrap analysis (100 replicates) [21].

The nonsynonymous/synonymous rate ratio (dN/dS or ω) is a widely used method to detect positive selection. Positive selection is inferred when the rate of nonsynonymous (dN) substitutions is higher than that of synonymous (dS) substitutions (dN/dS >1).

To test for the action of episodic positive selection in flaviviruses, we applied the branch-site test [22] from the codeml software [23]. The test estimates selective pressure changes among branches and sites in the phylogenetic tree. Two nested models (MA and MA1) are compared: MA allows positive selection on one or more lineages (called foreground lineages), and the MA1 does not allow such positive selection. Twice the difference of likelihood for the two models (ΔlnL) is compared to a χ^2^ distribution with one degree of freedom [22]. A false discovery rate correction was applied to take into account a multiple hypothesis issue generated by analyzing different branches on the same phylogeny [24].

A Bayes Empirical Bayes (BEB) analysis was used to evaluate the posterior probability that each codon belongs to the site class of positive selection on the foreground branch, only when 2ΔlnL was statistically significant.

BUSTED (branch-site unrestricted statistical test for episodic diversification) [25] is an alternative approach implemented in the HyPhy package [26] designed to describe episodic positive selection acting on specific branches in the phylogenetic tree at a proportion of sites. A model that allows the action on positive selection on foreground branches is compared with a null model that doesn't allow ω >1. Twice the ΔlnL of the two models is then compared to a χ^2^ distribution (with two degrees of freedom); if the null model is rejected, at least one site is under positive selection on the foreground branches. To detect selection at individual sites, twice the difference of the likelihood for the alternative and the null model at each site is compared to a χ^2^ distribution (one degree of freedom).

To be conservative, we considered a site under episodic positive selection if it showed both a *p* value ≤ 0.05 in BUSTED and a BEB posterior probability ≥ 0.90.

To better understand the evolution of ZIKV genomes, we also applied two random site (NSsite) models implemented in codeml: a null model (M7) that assumes that 0<ω<1 and is beta distributed among sites in all branches of the phylogeny, and a positive selection model (M8); this model is the same as M7 but also includes an extra category of sites in the alignment with ω>1. A χ^2^ distribution is used to assess statistical significance of 2ΔlnL of the two models.

Positively selected sites were identified using the posterior probability (≥ 0.90) from M8 BEB.

Individual sites under diversifying positive selection were also identified using Random effects likelihood (REL) [27] and Fast Unconstrained Bayesian AppRoximation (FUBAR) [28] methods from the HyPhy package.

REL estimates ω at each site by inferring a gene distribution for both synonymous and non-synonymous rate variations and assuming independent draw at each site from this distribution. We considered a site under positive selection if it showed a Bayes Factor > 50.

FUBAR is an approximate hierarchical Bayesian method that uses an unconstrained distribution of selection parameters by averaging over a large number of predefined site classes. Given this distribution, FUBAR estimates the posterior probability of positive diversifying selection at each site in the alignment (with a cutoff ≥ 0.90).

In order to be conservative, we finally considered a site as under diversifying positive selection if it was detected by at least two different methods.

### Immune epitopes

Data on DENV experimentally verified immune epitopes were obtained from the NIAID Virus Pathogen Database and Analysis Resource (ViPR) online (http://www.viprbrc.org) [29]. Human epitopes were searched for by using the gene product name as a query. Linear epitopes with positive results in any assay type category (B cell, T cell and MHC binding) were included. We used ClustalOmega [30] to align epitopes onto the DENV protein sequence and from this onto the ZIKV sequence.

### Protein 3D structures, homology modeling, and membrane topology

The structure of NS1 of ZIKV was obtained by homology modeling using the West Nile virus NS1 (PDB ID: 4O6C) structure as a template; analysis was performed through the SWISS-MODEL server [31]. The accuracy of the model was assessed with VADAR (Volume, Area, Dihedral Angle Reporter), which uses several algorithms to calculate different parameters for individual residues and for the entire protein [32].

Images were created using PyMOL (The PyMOL Molecular Graphics System, Version 1.5.0.2 Schrödinger, LLC).

The membrane protein topology for the ZIKV NS4B protein was predicted by using TMHMM (http://www.cbs.dtu.dk/services/TMHMM/) [33].

## Results

### NS1 is the major target of positive selection during flavivirus speciation

As mentioned above, ZIKV belongs to the Spondweni group of mosquito-borne flaviviruses. In addition to Spondweni virus (SPOV), the viral species more closely related to ZIKV include the Kedougou (KEDV) and dengue (DENV) viruses [10]. To investigate selective events that took place during the speciation of ZIKV and closely related flaviviruses, we obtained complete coding sequence information for 21 ZIKV, 1 SPOV and 1 KEDV, as well as 11 DENV. ZIKV sequences were selected to represent viruses sampled in both African and in non-African countries, in distinct years, and from different hosts (see S1 Table). In the case of KEDV and SPOV, only one complete genome is available for each virus. For DENV, sequences were selected to belong to the four major serotypes (DENV1 to DENV4); for each serotype, sequences representative of the most common genotypes (based on complete E nucleotide sequences in [34]) were included. DENV sequences were also selected to cover different geographic locations and isolation dates.

The structural and nonstructural coding regions were analyzed separately, and the alignments were pruned of unreliably aligned codons using the GUIDANCE utility (see Methods). This procedure resulted in the masking of 8.6% and 9.5% of codons in the structural and nonstructural regions, respectively. A test for substitution saturation was performed using Xia's method [16] and indicated no substantial saturation in either alignment (S2 Table).

**Fig 1:**
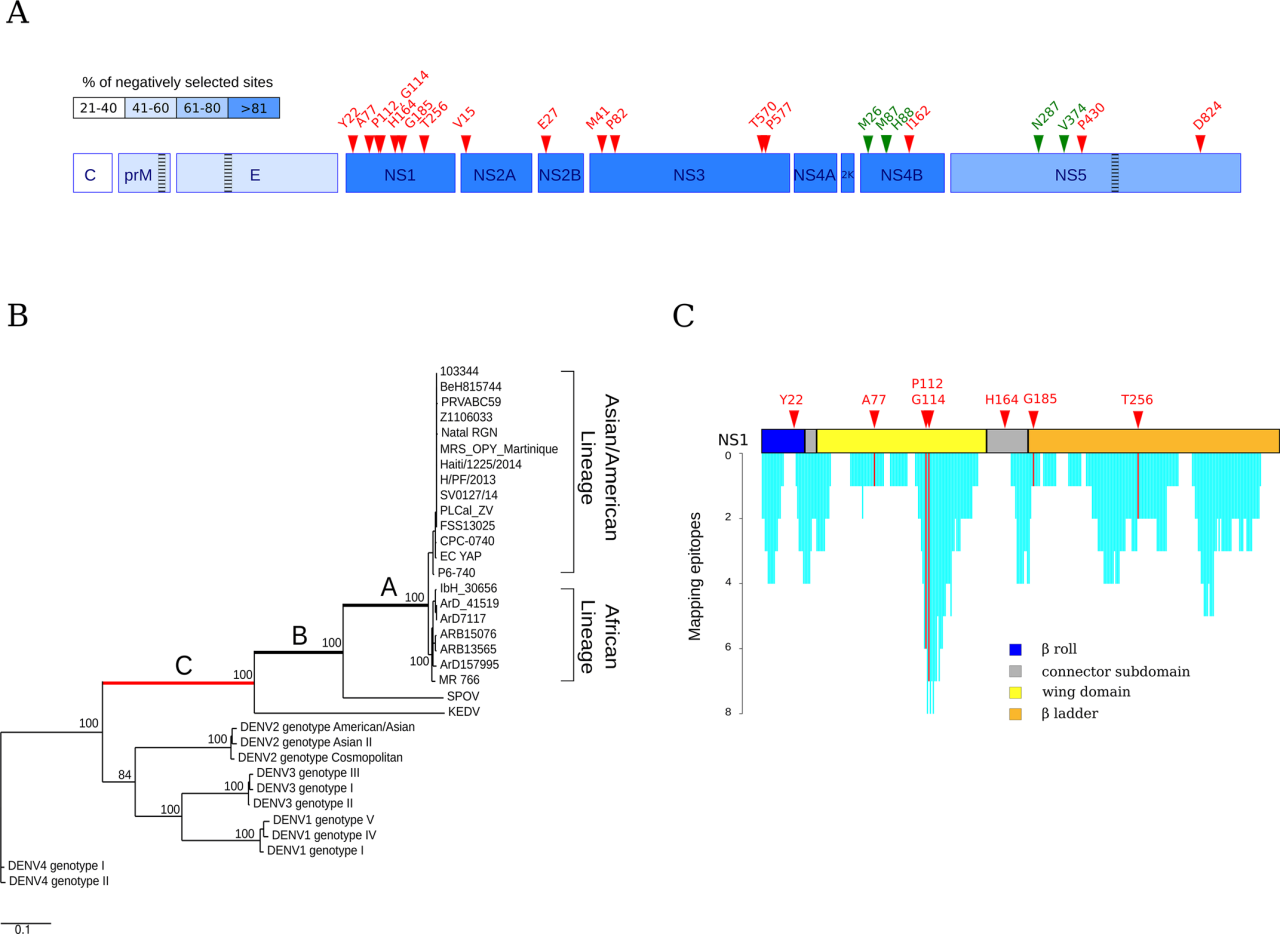
Different selective pressures acting on flavivirus polyproteins. (A) Schematic representation of the ZIKV polyprotein. Proteins are colored in hues of blue depending on the percentage of negatively selected sites in ZIKV strains. The location of recombination breakpoints in flaviviruses and ZIKV is shown by striped rectangles. Positively selected sites in the flavivirus phylogeny and in ZIKV strains are colored in red and green, respectively. (B) Maximum likelihood unrooted tree for the flavivirus phylogeny. Branches analyzed in the branch-site tests are indicated with capital letters, with red indicating statistically significant evidence of positive selection. Branch length is proportional to nucleotide substitutions per codon. Bootstrap values for internal branches >75% are shown. See S1 Table for accession number and full names of analyzed viruses. (C) Immune epitope mapping and schematic representation of NS1 domains. Cyan bars indicate the number of epitopes overlapping each NS1 residue. Positively selected sites are also shown.

We next analyzed the alignments for the presence of recombination using two different methods, GARD (Genetic Algorithm Recombination Detection) [18] and Recco [19]. No evidence of recombination in the nonstructural region was detected using either program, whereas Recco (but not GARD) suggested the presence of a recombination breakpoint around position 1350–1360 (relative to AY632535 coding sequence) in the E region (Fig 1A). The structural region was thus split into two sub-regions for the following analyses, so as to avoid false positive inferences of positive selection as a result of unaccounted recombination [35].

Phylogenetic trees of the three regions were obtained with phyML. Trees were very similar and fully consistent with previously reported phylogenies for flaviviruses [36], with African and non-African ZIKV isolates forming distinct branches [3] (Fig 1B).

We next searched for evidence of episodic positive selection along the internal branches of flavivirus phylogenies using branch-site tests (Fig 1B). Two different methods were applied to ensure consistency: the branch-site unrestricted statistical test for episodic diversification (BUSTED) [25] and the maximum-likelihood models (MA/MA1) implemented in the PAML suite [23]. These two approaches rely on different assumptions of ω (nonsynonymous/synonymous rate ratio) variation among branches. Episodic positive selection at each tested branch was declared when statistically significant support was obtained with both methods. Using this criterion, we found no evidence of positive selection in the structural region. Conversely, both tests detected evidence of positive selection on one branch in the phylogeny of the nonstructural region (Fig 1 and Table 1). Selected sites along this branch were identified using the Bayes empirical Bayes (BEB) procedure from model MA and with BUSTED; again, only sites detected by both methods were considered. A total of 16 positively selected sites were detected (Fig 1A); notably, seven of such sites are located in the NS1 protein. To test whether this number is higher than expected, we performed random sampling across codons in the nonstructural region (i.e. we assumed that all codons that were not masked by GUIDANCE in any sequence have the same probability of being called as positively selected). Results indicated that the likelihood of having 7 selected sites in NS1 amounts to 0.0039; thus, this protein represented a preferential selection target during flavivirus speciation.

**Table 1:** Branch-site analyses of the flavivirus polyprotein (nonstructural region).

	MA vs MA1		BUSTED	
Foreground Branch^a^	-2ΔlnL^b^	*p* value (Bonferroni corrected *p* value)	-2ΔlnL^b^	*p* value (Bonferroni corrected *p* value)
Branch A	22.00	2.73x10^-6^ (8.19x10^-6^)	0.32	1 (1)
Branch B	35.22	2.94x10^-9^ (8.83x10^-9^)	4.36	1 (1)
Branch C	34.24	4.87x10^-9^ (1.46x10^-8^)	8.10	0.016 (0.048)

^**a**^ Branches are named as in Fig 1

^**b**^ Twice the difference of likelihood for the null and the alternative models compared.

We note that the percentage of codons masked by GUIDANCE ranged widely among protein regions, from 3.3% in NS3 to more than 30% in the 2k and NS2A region (S3 Table). Whereas this does not affect the significant enrichment we obtained for NS1 (as we accounted for masked codons), the power to detect selection in extensively masked regions is clearly reduced; in fact, the ultimate result of masking is fewer codons available for analysis or a shallow phylogeny at available codons.

**Fig 2:**
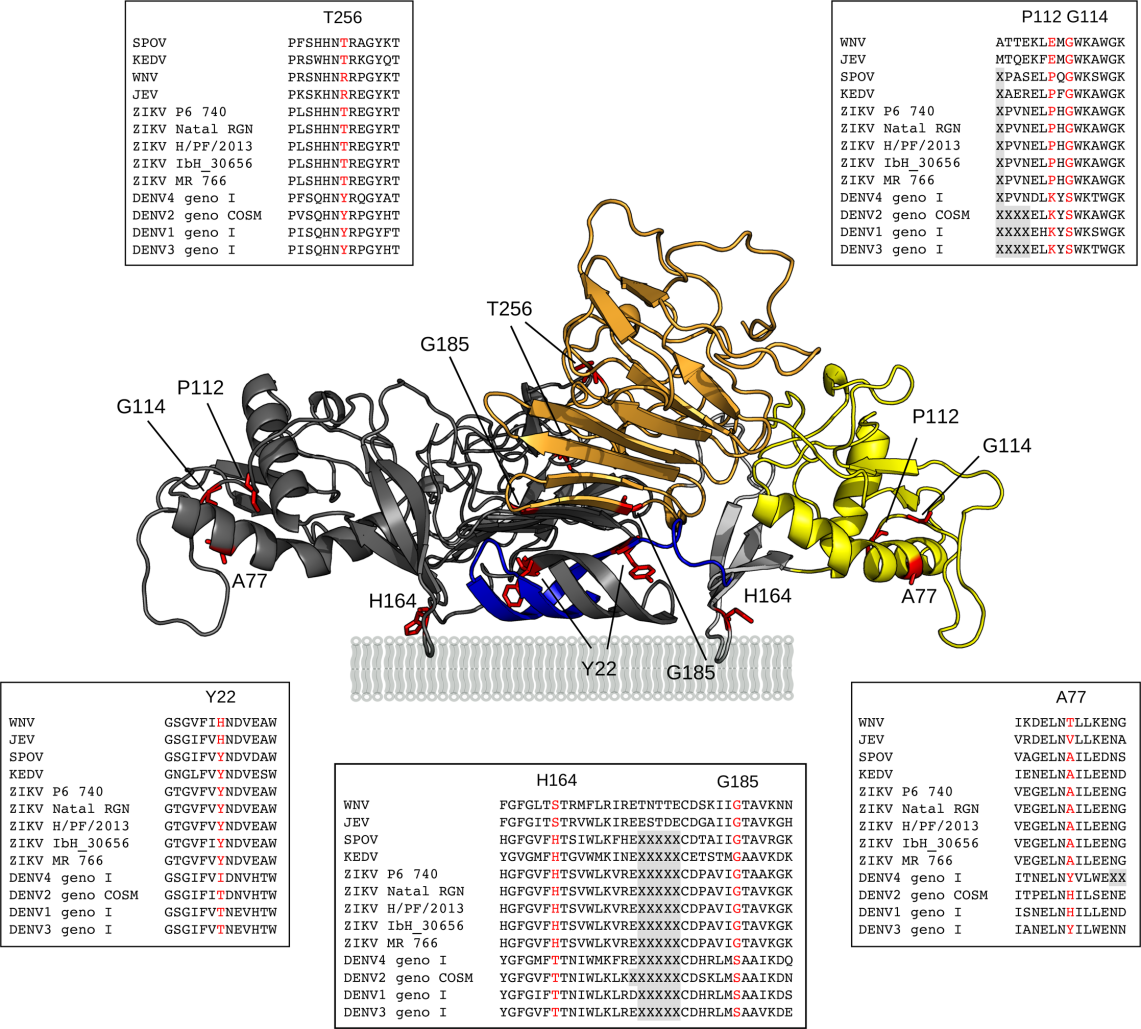
3D mapping of NS1 positively selected sites. 3D structure of ZIKV NS1 dimer obtained by homology modeling of WNV NS1 (PDB ID: 4O6C). Domain colors are as in Fig 1C. Positively selected sites are shown in red. Protein alignments of the regions surrounding selected sites are also shown for a few representative flavivirus species (JEV, NC_001437; WNV, NC_001563). Amino acids corresponding to codons filtered by GUIDANCE are represented by shaded X letters.

In NS1, the 7 positively selected sites are distributed along the entire protein region (Fig 1C). To gain insight into the role and the spatial localization of these sites, we performed homology modeling of ZIKV NS1 using the West Nile virus protein structure (PDB ID: 4O6C) as a template (Fig 2). We also retrieved the location of experimentally validated immune epitopes in NS1. Selected sites 112 and 114 map on a disordered loop of the wing domain; this loop is exposed, and several DENV immune epitopes were described in this region, most of them covering both positions (Fig 1C). In DENV, an alanine mutation at the 114 site affects virus particle production [37]. This residue was also suggested to have a role in the interaction between NS1 and the envelope glycoprotein [37]. Interestingly, three sites (residues 77, 112, 114) in the “wing” domain localize in close proximity on the protein structure (Fig 2), suggesting that they are involved in formation/stabilization of the same interactions. NS1 position 164 is located in a hydrophobic protruding loop, flanking a smaller loop that is essential for DENV viral replication [38]. Mutations in flanking positions (residues 160 and 162) affect both RNA synthesis and virus viability [38]. Indeed, residues 159–162 of the connector domain together with the β-roll (where the Y22 selected site maps) form a hydrophobic protrusion that faces the membrane (Fig 2). This hydrophobic structure is thought to be involved in the interaction between the NS1 homodimer and the replication complex through the NS4A and NS4B proteins [38]. The β-roll domain is also involved in NS1 dimerization [38]. Site 22 localizes in spatial proximity to the first β-strand of the β-ladder domain, where the G185 positively selected site also maps. Both sites are located at the dimerization interface [38, 39].

As for selected sites in proteins other than NS1, site V15 in NS2A maps to a hydrophobic protein region within the lumen of the endoplasmic reticulum (ER); mutations at nearby residues impair DENV virion assembly [40]. Positively selected sites were also detected in NS3 (M41, P82, T570, P577). Interestingly, site 570 flanks a conserved asparagine that is essential for NS3-NS5 binding in DENV [41]. Finally, the positively selected site in NS4B (I162) is located in a cytoplasmic loop involved in the interaction with NS3 and with host proteins [42].

### Ongoing positive selection at nonstructural proteins in ZIKV

We next investigated whether positive selection also occurred during the recent evolution of ZIKV. To this purpose, we retrieved coding sequence information for all complete ZIKV genomes (n = 39, as of March 26^th^, 2016) (S1 Table). Again, the structural and nonstructural regions were analyzed separately. In the structural region, GARD detected no recombination, whereas Recco inferred possible breakpoints at nucleotides 802–838 (relative to AY632535 coding sequence) in the M region. Both Recco and GARD detected a recombination breakpoint in the nonstructural region (position 8994, GARD; position 9040–9054, Recco) within the NS5 region. The portions encompassing the breakpoint positions were thus removed and the alignments were split into two sub-regions. Inspection of the Recco output indicated that in all cases recombination involved sequences from the African isolates. In fact, the phylogenetic trees for all sub-regions showed a clear separation of the African and non-African sequences (S1 Fig).

To obtain an estimate of the degree of constraint along ZIKV genomes, we used FUBAR to identify sites showing significant evidence of negative selection. This analysis indicated an uneven distribution of negatively selected sites, with the weakest selective pressure acting on the structural portion; conversely, more than 80% of sites are negatively selected in the NS1-NS4B region (Fig 1A).

**Fig 3:**
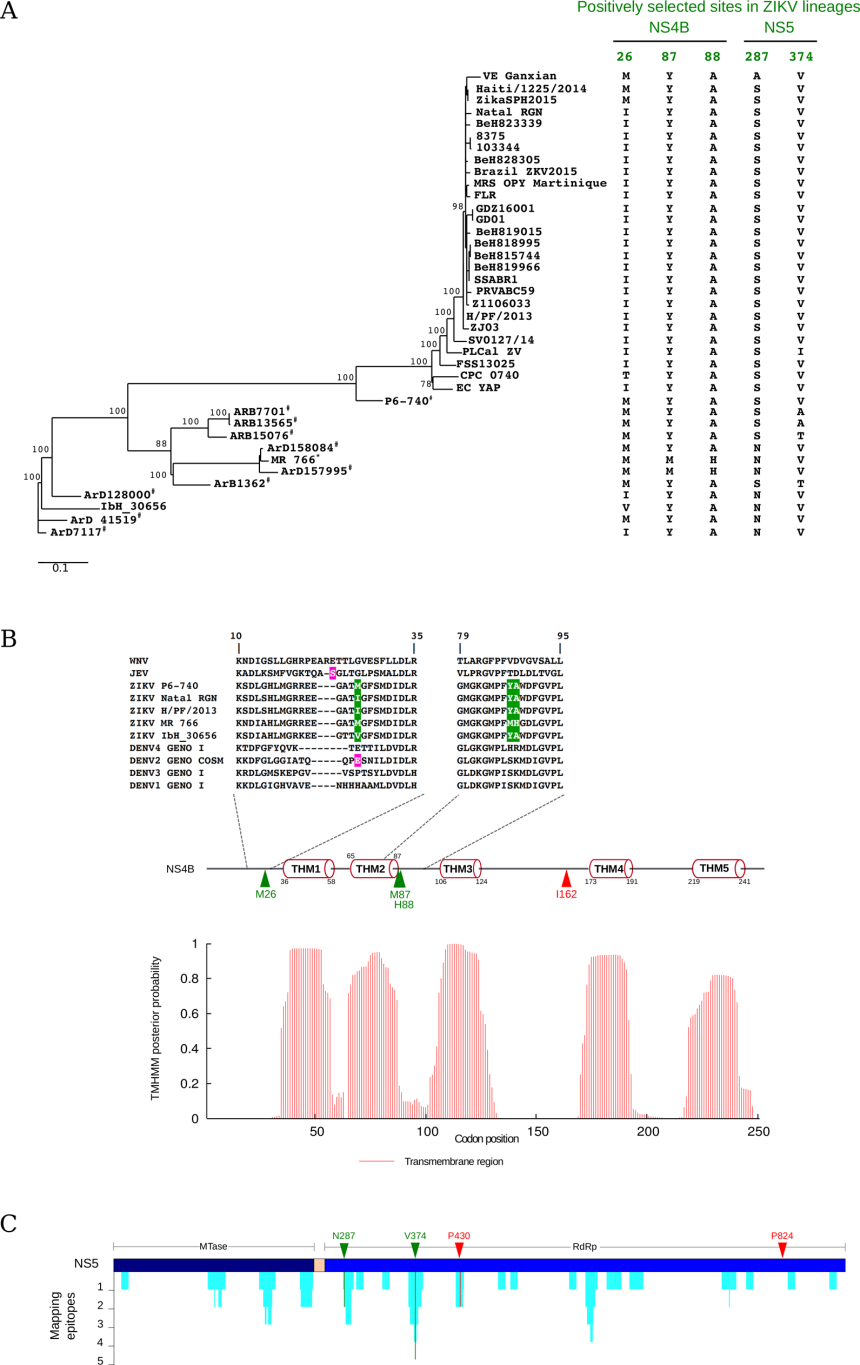
Ongoing positive selection in ZIKV isolates. (A) Maximum likelihood phylogeny for the nonstructural region (nucleotides 2371 to 8994). Amino acids at the five selected sites are shown for all ZIKV sequences. Viruses isolated from mosquitos and macaques are denoted with hash and asterisk symbols, respectively. Branch length is proportional to nucleotide substitutions per codon. Bootstrap values for internal branches >75% are shown. The phylogenetic tree is unrooted. (B) TMHMM prediction of transmembrane helices (TMH1-5) for the ZIKV NS4B protein and schematic representation of protein topology. Positively selected sites in the flavivirus phylogeny and in ZIKV strains are indicated by red and green triangles, respectively. Amino acid alignments of the regions surrounding selected sites are shown for 5 representative ZIKV, for DENV sequences belonging to the four serotypes, for JEV (NC_001437), and for WNV (strain NY-99, NC_001563). In the alignment, positively selected sites in ZIKV are shown in green; sites that are positively selected in other flaviviruses are marked in magenta. (C) Immune epitope mapping on NS5. Cyan bars indicate the number of epitopes overlapping each NS5 residue. Positively selected sites are colored as above. Mtase: methyltransferase domain; RdRp: RNA-dependent RNA polymerase domain.

We next tested for positive selection using both the codeml site models (M7 vs M8) and the branch-site models (MA1 vs MA). These latter models were used to test for selection on the branch of the phylogeny that separates the African and non-African sequences. No evidence of positive selection was obtained for the two sub-regions from the structural portion. Conversely, for the nonstructural region covering nucleotides 2371–8994, a model of evolution that allows a class of codons to evolve with ω >1 (NSsite model M8) better fitted the data than the neutral model (NSsite models M7), supporting the action of positive selection (-2ΔlnL = 18.89, degrees of freedom = 2, Bonferroni- corrected *p* value for two tests = 1.58 X 10^−4^). Positively selected sites were identified using the BEB procedure from M8 and with two additional methods from the HyPhy suite, REL and FUBAR. Sites were defined as being positively selected if they were detected by at least two different methods. Using this conservative criterion, 5 positively selected sites were detected (Table 2 and Figs 1A and 3). Three of them are located in the relatively short NS4B region (M26, M87, and H88); using the same approach as above, we determined that this number is unlikely to occur by chance (random sampling, *p* value = 0.007), indicating that NS4B is the preferential positive selection target in ZIKV.

**Table 2:** Positively selected sites in ZIKV polyprotein.

Position^a^	Protein	BEB pp^b^	REL Bayes Factor	FUBAR pp^b^
26	NS4B	0.92	1117.62	0.76
87	NS4B	0.99	2049.42	0.34
88	NS4B	0.94	187.395	0.20
287	NS5	0.89	526.902	0.91
374	NS5	0.96	129.954	0.23

^a^ Relative position in protein

^b^Posterior Probability

To gain insight into the location of positively selected sites in NS4B, we performed an *in silico* prediction of transmembrane helices. The resulting topology model was very similar to those previously proposed or determined for other flaviviruses [42] (Fig 3B). Residue M26 maps to the N-terminal region located in the ER lumen. Interestingly, the corresponding position was previously found to be positively selected in sylvatic DENV2 isolates; in JEV, a nearby residue is positively selected, as well [43, 44] (Fig 3B). Residues 87 and 88 are also located in the ER lumen and reside in the second loop (Fig 3B), a region involved in NS4B-NS1 interaction in WNV [45].

Two other positively selected sites are located in NS5 (N287 and V374). Position 374 is part of the nuclear localization signal (NLS) region of NS5. Dengue virus serotypes have different nuclear localization, and these differences are due to changes in their NLS [46]. Analysis of DENV immune epitopes indicate that some of them comprise positions 374 and 287 (Fig 3C).

## Discussion

Herein we provide an analysis of the selective forces acting on ZIKV and related flaviviruses. We show that positive selection contributed to the genetic diversity of these human pathogens and we report ongoing adaptive evolution in ZIKV strains.

The evolutionary analysis of viral genomes poses challenges related to the possible presence of recombination, as well as to the high sequence divergence, with consequent saturation issues and alignment uncertainties. We accounted for all these possible confounding effects, which would otherwise affect inference of positive selection. Indeed, we adopted recommended alignment and filtering criteria to minimize erroneous codon alignments [15], and we tested for substitution saturation. As for recombination, we applied two methods, based on different features of the data, to screen the alignments and to infer the most likely position of breakpoints. These latter were used to split alignments into sub-regions that were separately analyzed. In this respect, it is worth noting that we did not detect recombination breakpoints in the nonstructural region for the extended flavivirus phylogeny, whereas we found evidence of recombination when all ZIKV strains alone were analyzed. The explanation for this apparently contradictory finding is that one single ZIKV African sequence contributed to the recombination events and it was not represented in the flavivirus phylogeny. Moreover, the flavivirus alignment was partially masked to remove unreliably aligned codons. This procedure clearly determines the removal of the most divergent regions, which may derive from recombination events. This most likely accounts for the discrepancy between our results and those from a previous report that indicated recombination between Asian ZIKV strains and SPOV within NS2B [47]. Another previous study analyzed African and non-African ZIKV isolates and reported the presence of four recombination breakpoints in ZIKV genomes [48]. In our analysis we only detected two breakpoints. We believe that the main reason for the discrepancy with this previous analysis derives from the fact that the ArD142623 strain, which contributed most recombination events in Faye's dataset [48], was not included in our study because its genome sequence is not complete and because its polyprotein sequence is annotated as “nonfunctional due to mutation” in GenBank. In this respect, it is worth mentioning that despite our findings and those previously reported by others for ZIKV [47, 48] and DENV [34], experimental data have indicated that flaviviruses have very low propensity for recombination [49, 50]. Moreover, under laboratory conditions specifically devised to detect recombination, extremely rare events were observed that generated aberrant JEV genomes with reduced growth properties [50]. These observations raise the possibility that recombination events identified through analysis of existing sequences in public databases are artifacts of laboratory contamination or incorrectly assembled sequence files. This was previously suggested to be the case for some “recombinant” DENV sequences [34, 51]. All the recombination events we detected involved one or few sequences from African ZIKV isolates. Whereas we cannot control for the accuracy of the deposited sequences, we have to take the possible recombination events into account; failure to do so would affect positive selection inference, irrespective of whether recombination actually occurred. Clarification of these potential caveats, though, is extremely relevant for epidemiological and preventive purposes. Because ZIKV, DENV, and other arboviruses can co-circulate during outbreaks [10], it will be extremely important to assess if and with what efficiency these viruses can recombine.

The branch-site tests we applied to analyze the flavivirus phylogeny were aimed at detecting episodic positive selection—i.e. selective events on one branch of the phylogeny and thus likely to have occurred during or after speciation. Using this approach we were able to show that positive selection acted on the branch that separates DENV from the other analyzed flaviviruses and mainly targeted NS1. It should be noted that the branch-site tests have low false positive rates and are largely insensitive to violations of the assumption of neutral evolution on the background branches [22, 24], but lack power [52]. Thus, selection may act on additional branches than the one we detected and more selected sites may exists. When analysis was performed on ZIKV genomes, which are characterized by much lower divergence compared to the flavivirus sequences in the inter-species analysis, tests to detect episodic and pervasive positive selection were applied. The branch-site test showed no evidence of episodic selection and we consequently identified no selective events leading to the spread of the Asian ZIKV lineage. We mention, however, that the branch-site test may have failed to detect weak selection or selection at a very limited number of codons. We also stress that the lack of selection signatures does not imply that amino acid differences between African and non-African ZIKV lineages are irrelevant or nonfunctional.

Conversely, we identified pervasive selection—i.e. selective events that involve all ZIKV lineages- and, again, selected sites were found to occur in nonstructural portions of the ZIKV genome (NS4B and NS5). These portions also display the strongest level of selective constraint.

Structural proteins (the E protein in particular) might be a priori considered to be preferential selection targets during flavivirus evolution for least two reasons: these proteins [1] mediate the initial and essential steps of host infection via host cell binding and entry and [2] represent major targets for immune responses influencing antigenic selection [53]. Nevertheless, we found no evidence of positive selection in structural regions, either in flaviviruses or in ZIKV isolates. To our knowledge, no study has investigated the occurrence of positive selection in ZIKV or during flavivirus speciation, but efforts at detecting positive selection in DENV strains or isolates were performed. Depending on the serotype analyzed, on the geographic and temporal origin of the viruses, as well as on their transmission cycle (sylvatic or endemic), different genomic regions were found to represent targets of positive selection in DENV [54–59]. These regions were not limited to the structural portion, but also included nonstructural proteins [54–58]. Likewise, analysis of JEV sequences revealed selection in both structural and nonstructural regions [43, 44]. Also, ample evidence indicated that although most neutralizing antibodies are directed against flavivirus E proteins [53], non-neutralizing anti-NS1 antibodies are protective against severe disease during DENV, YFV, and JEV infection [60–64].

Finally, cell-mediated immunity was shown to target both structural and nonstructural DENV proteins, with the vast majority of T-cell epitopes located in nonstructural proteins [65]. In this respect, it is worth noting that several of the positively selected sites we detected in NS1 and in NS5 are located within immune epitopes. This observation suggests that the underlying selective pressure responsible for selection at these sites is exerted by the host adaptive immune system. These data are likely to be relevant for the current efforts to develop a ZIKV vaccine and to assess the possible cross-protection afforded by natural or vaccine-induced immunity against other related viruses.

Nonstructural proteins play different roles in flavivirus life cycles and several of them interact with innate immunity molecules. NS1, the major selection target in the flaviviruses we analyzed herein, is essential for viral RNA replication and is involved in immune system evasion. In particular, secreted hexameric NS1 represents a major antigenic marker of viral infection for all DENV serotypes. Soluble NS1 in the serum of patients has been found to correlate with severe clinical disease [66], suggesting that the NS1 protein also plays an important role in the pathogenesis of dengue. Importantly, the NS1 protein from WNV and DENV2 interacts with multiple components of the complement system (C1S, C4, C4-binding protein, CFH), as well as with toll-like receptors (TLR3, TLR2, and TLR6) [67, 68]. The molecular details of these interactions are presently unknown, but the presence of several positively selected sites in NS1 suggests a possible arms race with the host innate immune system. It will be extremely important to assess whether amino acid differences in flavivirus NS1 proteins affect the interaction with innate immunity components and consequently modulate the host response to ZIKV or DENV.

NS1 is also required for efficient viral genome replication. Recently, it has been proposed that dimeric NS1 plays an organizational role in the formation of the replication complex on the cytoplasmic side of the ER membrane [69], and that this function is mediated by interactions with NS4A and NS4B [45, 70]. Interestingly, the positively selected site in the connector domain (residue 164) is located in the hydrophobic protrusion that may contact NS4A and NS4B [38]. Although mutation of residue T164 to alanine has no effect on RNA replication or on the assembly of DENV particles [37], we cannot exclude its involvement in (de)stabilizing the interaction with the ER membrane; in fact, the introduction of a histidine (Fig 2) at this site might affect the protein function more importantly than the conservative alanine substitution. Scaturro and colleagues [37] also reported that NS1 plays a critical role in the biogenesis of DENV virions, a function that is mediated by interaction with structural proteins. In this context, a key role is played by two residues (114 and 115) in the flexible loop of the NS1 wing domain. Indeed, alanine mutation of residue S114 abrogates DENV2 NS1 binding to E, prM, and C [37]. Notably, we identified residue 114 as positively selected in the flavivirus phylogeny and two additional selected sites were located in close spatial proximity.

Overall, these observations suggest that positive selection at NS1 is acting to optimize viral fitness by modulating viral replication efficiency and/or favoring evasion from the host immune system. Similar considerations may apply to NS4B, which displays 3 positively selected sites in ZIKV isolates. This membrane protein has a role in the formation of the replication complex and in virus pathogenesis [71]. Several mutations in the NS4B region of JEV, YFV, and WNV were shown to modulate neurovirulence and/or neuroinvasiveness [42]. Notably, one of the positively selected sites we identified in NS4B (26M/I/T/V) was previously reported to represent a selection target in sylvatic DENV2 isolates but not in the endemic strains [57]. A nearby residue was also found to evolve adaptively in JEV, both in genotype I and genotype III isolates [43, 44]. However, variation at this site seems not to be associated with host preferences in JEV [44]. Although the functional significance of changes at position 26 in NS4B remains to be clarified, the fact that this residue or a flanking one is targeted by positive selection in three closely related flaviviruses suggests an important role in viral adaptation. Indeed, the N-terminal region of flavivirus NS4B (amino acids 1–125) inhibits interferon (IFN) response by blocking IFN-α/β signaling [72]. This region includes two additional positively selected sites (M87 and H88) and is involved in host protein (e.g. STING) binding. It is interesting to note that YFV NS4B, but not DENV NS4B, can bind STING [73], suggesting that positive selection in this region results from adaptation to the host innate immune system to modulate binding of viral sensors. The region surrounding positions 87 and 88 is also responsible for NS1-NS4B binding and the same study demonstrated the importance of the F86C mutation in WNV NS4B to rescue viral replication in presence of NS1 nonfunctional mutations [45]. Finally, the DENV NS4B region spanning residues 84 to 146 is required for interaction with NS4A, another molecule involved in flavivirus replication [74].

Thus, based on data from other flaviviruses, the three positively selected sites we identified in NS4B of ZIKV are located in a protein region important for interaction with other viral proteins and with host molecules.

The relatively sparse sampling of ZIKV genomes and the paucity of ZIKV sequences isolated from humans in Africa and from mosquitoes in Asia/America, prevents drawing any definite conclusion about the role of selected sites on host preference, pathogenicity, or infectivity. Moreover, as anticipated above, a potential issue associated with viral sequence analysis concerns laboratory contaminations, especially during serial passages in culture. Contaminations were previously suggested to account for discrepancies in DENV phylogenies [34], and a few of the African ZIKV strains we included in the study were passaged several times in suckling mice or cell culture [9]. This process may also introduce variants that are not present in nature, potentially affecting evolutionary inference. These issues are unlikely to affect the analyses we performed on the extended flavivirus phylogeny, as variation on terminal branches has minor effects. However, these caveats should be kept in mind in the analysis of ZIKV strains, especially for positively selected sites showing variability in a minority of sequences. Further evolutionary analysis of ZIKV will greatly benefit from the sequencing and inclusion of additional isolates, not only from the ongoing American epidemic, but also from African countries.

Despite these limitations, we suggest that the positively selected sites we identified should be prioritized in future experimental studies. These amino acids changes are expected to modulate aspects of viral fitness, either in mosquitoes or vertebrate hosts. In this respect, reverse genetic approaches will be instrumental to assess the role of specific changes on different viral phenotypes including transmission by distinct Aedes mosquito vectors or alternative (e.g. human-to-human) transmission modes, increased viremia in humans, and altered tissue tropism.

Finally, we note that NS1 and NS4B are regarded as attractive candidates as direct or indirect targets for antiviral drugs in flavivirus infections [42, 75]. Nonetheless, these proteins are fast evolving in ZIKV and related flaviviruses, and the numerous selected sites are expected to entail functional differences among closely related viruses or even among viruses belonging to the same species. Thus, our data suggest that compounds developed against DENV NS4B [42] or drugs that result in DENV NS1 misfolding [75, 76] may not be active against ZIKV.

## Supporting Information

S1 Fig Effect of recombination on tree topology. Maximum likelihood phylogeny for two subregions of ZIKV non-structural region based on the recombination breakpoint. Branch length is proportional to nucleotide substitutions per codon. The phylogenetic trees are unrooted. Bootstrap values for internal branches >75% are shown.
(PDF)

S1 Table List of viral sequences. (PDF)

S2 Table Substitution saturation analysis of flavivirus polyprotein. (PDF)

S3 Table Percentage of GUIDANCE-masked codons in each protein region. (PDF)
