# Supplementary figures and images for: Nonstructural Proteins Are Preferential Positive Selection Targets in Zika Virus and Related Flaviviruses
Source: PLoS Negl Trop Dis. 2016 Sep 2;10(9):e0004978. doi: 10.1371/journal.pntd.0004978 (PMC5010288; doi:10.1371/journal.pntd.0004978)

REGION 1

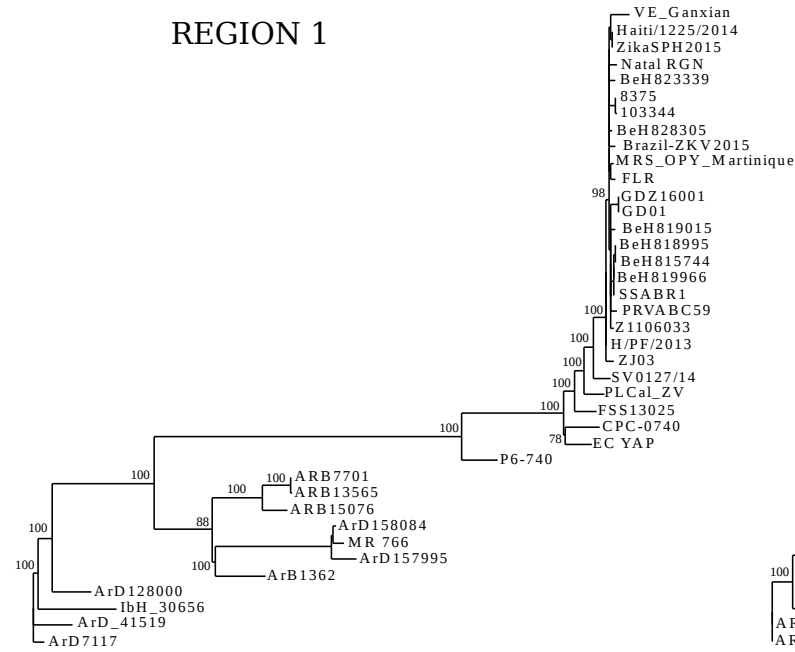

0.1

REGION 2

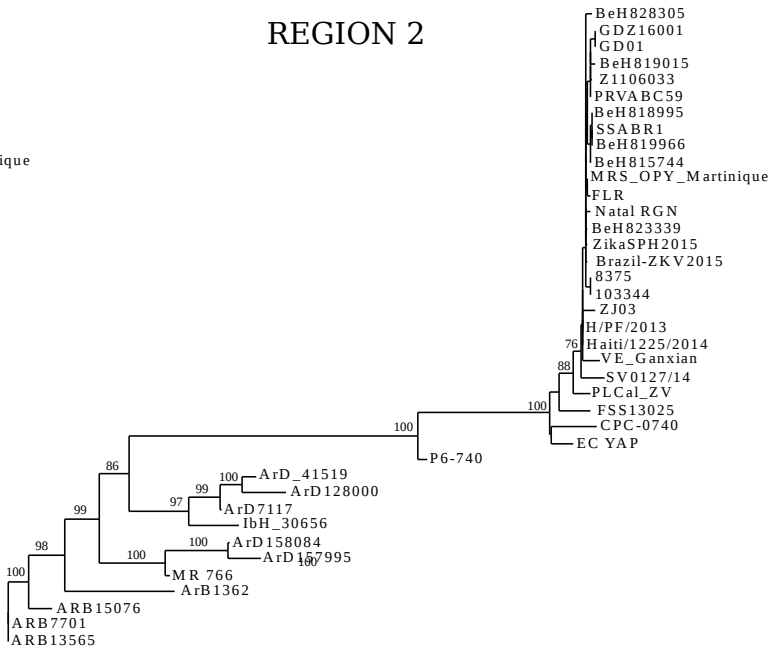

0.1

Supplement: S1 Fig — Maximum likelihood phylogeny for two subregions of ZIKV non-structural region based on the recombination breakpoint. Branch length is proportional to nucleotide substitutions per codon. The phylogenetic trees are unrooted. Bootstrap values for internal branches >75% are shown. (PDF) [file pntd.0004978.s001.pdf]
