# Supplementary material for: Nonstructural Proteins Are Preferential Positive Selection Targets in Zika Virus and Related Flaviviruses
Source: PLoS Negl Trop Dis. 2016 Sep 2;10(9):e0004978. doi: 10.1371/journal.pntd.0004978 (PMC5010288; doi:10.1371/journal.pntd.0004978)
Supplement: S1 Table — (PDF) [file pntd.0004978.s002.pdf]

S1 Table. List of viral sequences.

Species	NCBI Accession Number	Strain Name (genotype)	Country	Host	Collection Date
ZIKV	KU365778	BeH819015	Brazil	*Homo sapiens*	2015
ZIKV	KU365777	BeH818995	Brazil	*Homo sapiens*	2015
ZIKV	KU501217	8375	Guatemala	*Homo sapiens*	2015
ZIKV	KU501216	103344*	Guatemala	*Homo sapiens*	2015
ZIKV	KU501215	PRVABC59*	Puerto Rico	*Homo sapiens*	2015
ZIKV	KU647676	MRS_OPY_Martinique_PaRi_2015*	Martinique	*Homo sapiens*	2015
ZIKV	KU681082	CPC-0740*	Philippines	*Homo sapiens*	2012
ZIKV	KU681081	SV0127/14*	Thailand	*Homo sapiens*	2014
ZIKV	KU707826	SSABR1	Brazil	*Homo sapiens*	2015
ZIKV	KU527068	Natal RGN*	Brazil	*Homo sapiens*	2015
ZIKV	KU509998	Haiti/1225/2014*	Haiti	*Homo sapiens*	2014
ZIKV	KU820899	ZJ03	China	*Homo sapiens*	2016
ZIKV	KU820897	FLR	Colombia	*Homo sapiens*	2015
ZIKV	KU729217	BeH823339	Brazil	*Homo sapiens*	2015
ZIKV	KU729218	BeH828305	Brazil	*Homo sapiens*	2015
ZIKV	KU761564	GDZ16001	China	*Homo sapiens*	2016
ZIKV	KU744693	VE_Ganxian	China	*Homo sapiens*	2016
ZIKV	KU740184	GD01	China	*Homo sapiens*	2016
ZIKV	KU497555	Brazil-ZKV2015	Brazil	*Homo sapiens*	2015
ZIKV	EU545988	EC YAP*	Micronesia	*Homo sapiens*	2007
ZIKV	AY632535	MR 766*	Uganda	sentinel *Rhesus*	N.A.
ZIKV	HQ234499	P6-740*	Malaysia	*Aedes aegypti*	1966
ZIKV	HQ234500	IbH_30656*	Nigeria	*Homo sapiens*	1968
ZIKV	JN860885	FSS13025*	Cambodia	*Homo sapiens*	2010

ZIKV	KF268948	ARB13565*	Central African Republic	*Aedes africanus*	1976
ZIKV	KF268949	ARB15076*	Central African Republic	*Aedes opok*	N.A.
ZIKV	KF268950	ARB7701	Central African Republic	*Aedes africanus*	N.A.
ZIKV	KF993678	PLCal_ZV*	Canada	*Homo sapiens*	2013
ZIKV	KF383115	ArB1362	Central African Republic	*Aedes africanus*	1968
ZIKV	KF383116	ArD7117*	Senegal	*Aedes luteocephalus*	1968
ZIKV	KF383117	ArD128000	Senegal	*Aedes luteocephalus*	1997
ZIKV	KF383118	ArD157995*	Senegal	*Aedes dalzieli*	2001
ZIKV	KF383119	ArD158084	Senegal	*Aedes dalzieli*	2001
ZIKV	KJ776791	H/PF/2013*	French Polynesia	*Homo sapiens*	2013
ZIKV	KU321639	ZikaSPH2015	Brazil	*Homo sapiens*	2015
ZIKV	KU312312	Z1106033*	Suriname	*Homo sapiens*	2015
ZIKV	HQ234501	ArD_41519*	Senegal	*Aedes africanus*	1984
ZIKV	KU365780	BeH815744*	Brazil	*Homo sapiens*	2015
ZIKV	KU365779	BeH819966	Brazil	*Homo sapiens*	2015
DENV1	EU081262	DENV-1/SG/05K4173DK1/2005 (I)	Singapore	*Homo sapiens*	2005
DENV1	DQ285561	DENV-1 Seychelles 1480/04 (IV)	Seychelles	*Homo sapiens*	2004
DENV1	AF514889	DENV-1/AR/297arg00/2000 (V)	Argentina	*Homo sapiens*	2000
DENV2	AY776328	DENV-2/TW/Taiwan-1008DHF (Cosmopolitan)	Taiwan	N.A.	N.A.
DENV2	AY702034	DENV-2/CU/Cuba13-97/1997 (American/Asian)	Cuba	*Homo sapiens*	1997
DENV2	AF169681	DENV-2/TH/ThNH55-93/1993 (Asian II)	Thailand	*Homo sapiens*	1993
DENV3	AY744682	DENV-3/PF/PF92-2956/1992 (I)	French Polynesia	*Homo sapiens*	1992
DENV3	DQ675526	DENV-3/TW/98TW388/1998 (II)	Taiwan	*Homo sapiens*	1998
DENV3	AY679147	DENV-3/BR/BR74886-02/2002 (III)	Brazil	*Homo sapiens*	2002
DENV4	GQ868594	DENV-4/PH/BID-V3361/1956 (I)	Philippines	*Homo sapiens*	1956
DENV4	JN983813	DENV-4 Br246RR/10 (II)	Brazil	*Homo sapiens*	2010

KEDV	AY632540	DakAar D1470	Senegal	*Aedes spp.*	N.A.
SPOV	NC_029055	SM-6 V-1	South Africa	N.A.	N.A.

* ZIKV strains used in both flavivirus and ZIKV analyses.
ZIKV: Zika Virus; DENV: dengue Virus; KEDV: Kedougou Virus; SPOV: Spondweni Virus; N.A. Not Available.
