# Supplementary material for: Nonstructural Proteins Are Preferential Positive Selection Targets in Zika Virus and Related Flaviviruses
Source: PLoS Negl Trop Dis. 2016 Sep 2;10(9):e0004978. doi: 10.1371/journal.pntd.0004978 (PMC5010288; doi:10.1371/journal.pntd.0004978)
Supplement: S2 Table — (PDF) [file pntd.0004978.s003.pdf]

S2 Table. Substitution saturation analysis of flavivirus polyprotein.

Region	Number of Sequences[a]	Iss[b]	$I_{ss.cSym}$[c]	df[d]	*p* value[e]	$I_{ss.cAsym}$[f]	df[d]	*p* value[e]
Structural								
	4	0.481	0.838	1262	<0.0001	0.811	1262	<0.0001
	8	0.468	0.818	1262	<0.0001	0.722	1262	<0.0001
	16	0.485	0.801	1262	<0.0001	0.626	1262	<0.0001
	32	0.477	0.785	1262	<0.0001	0.512	1262	0.0062
Nonstructural								
	4	0.420	0.853	4258	<0.0001	0.845	4258	<0.0001
	8	0.462	0.846	4258	<0.0001	0.767	4258	<0.0001
	16	0.476	0.838	4258	<0.0001	0.681	4258	<0.0001
	32	0.468	0.813	4258	<0.0001	0.570	4258	<0.0001

[a] Number of species used in the random resampling.
[b] index of substitution saturation.
[c] critical value for a symmetrical tree topology.
[d] degrees of freedom.
[e] probability that I_{ss} is significantly different from the critical value ($I_{ss.cSym}/I_{ss.cAsym}$).
[f] critical value for an asymmetrical tree topology.

Note: two-tailed tests are used
