# Supplementary material for: Nonstructural Proteins Are Preferential Positive Selection Targets in Zika Virus and Related Flaviviruses
Source: PLoS Negl Trop Dis. 2016 Sep 2;10(9):e0004978. doi: 10.1371/journal.pntd.0004978 (PMC5010288; doi:10.1371/journal.pntd.0004978)
Supplement: S3 Table — (PDF) [file pntd.0004978.s004.pdf]

S3 Table. Percentage of GUIDANCE-masked codons in each protein region. We considered codons masked in at least one sequence.

	Protein region	Percentage
Structural	C	15.5
	prM	6.0
	E	7.9
Nonstructural	NS1	6.1
	NS2A	36.1
	NS2B	20.1
	NS3	3.3
	NS4A	7.1
	2K	54.2
	NS4B	12.9
	NS5	5.2
